# Family Medicine, the specialty of the future: the Portuguese situation within the European context

**DOI:** 10.1186/1755-7682-2-36

**Published:** 2009-11-11

**Authors:** Tiago Villanueva

**Affiliations:** 1 USF AlphaMouro, Algueirão-Rio de Mouro Health Centre Group, Lisbon, Portugal

## Abstract

General Practice/Family Medicine is a specialty focused on the provision of comprehensive, continuing, and community oriented, person-centred care. The lack of prestige and the difficulty in attracting trainees to the specialty have been longstanding problems in most countries around the world. In Europe, General Practice/Family Medicine is also hampered for not being recognized as a specialty throughout Europe. As for Portugal, General Practice/Family Medicine is undergoing a massive organizational reform, as well as unprecedented levels of popularity among trainees.

General Practice/Family holds tremendous latent potential, and is thus a specialty with a bright future ahead. It could well establish itself as the specialty of the future if it is able to overcome the barriers that currently make of General Practice/Family Medicine an unpopular career choice. It is important to train confident, competent and polyvalent family physicians, but it is also necessary to overhaul payment schemes, to invest in primary care infra-structure and organization, and to continue to attract more and more bright and motivated trainees.

## 

In the 24^th ^January 2009 issue of the Portuguese weekly newspaper "Expresso", Dr Luís Pisco, then President of the Portuguese Association of General Practitioners said: "today's freshers are the most brilliant students from their schools, and wish to pursue a technologically-driven specialty and make substantial amounts of money[1]."

This isn't a new phenomenon. General Practice has always lagged behind hospital specialties in terms of pulling power for the future generations of specialists.

In a recent article, Dr Juan Gérvas defined Primary Health Care as "all the accessible and comprehensive services available to citizens for the follow-up of their health problems. Specialists, Hospitals and emergency departments provide services focused on problems, whereas primary health care is focused on person-centred care[2]."

According to the European Society of General Practice/Family Medicine (WONCA Europe), "General practitioners/family doctors are specialist physicians trained in the principles of the discipline. They are personal doctors, primarily responsible for the provision of comprehensive and continuing care to every individual seeking medical care regardless of age, sex and illness[3]. "Core competencies of General practitioners/family doctors include primary care management, person-centred care, specific problem solving skills, comprehensive approach, community orientation, and a holistic approach[3].

The clinical work of general practitioners/family physicians includes health promotion, family health, prevention and treatment of acute diseases, treatment of chronic diseases, adult health, child health, women's health, mental health, rehabilitation and palliative care/terminal care[4]. In terms of technical expertise, the portfolio of general practitioners/family physicians varies between countries and includes almost everything, from placement of intrauterine devices to insertion of arterial lines[4].

Despite having a properly defined niche and purpose, General Practice/Family Medicine (GP/FM) suffers from lack of prestige. The causes of that lack of prestige include the contemporary culture of health consumerism and the triumph of the individual upon the population, the lack of awareness on behalf of the upper classes, a routine that is assumed to be too overburdened with trivialities and bureaucracy, the myth of a "primitive" and "non-scientific" medicine, and the low technological density[5].

Many European countries and other countries around the world are currently struggling with lack of general practitioners/family physicians, which is aggravated by the classic lack of popularity of the field among medical students and junior doctors. In Portugal, this lack of doctors is concentrated in the metropolitan areas of the largest cities, and Dr Luís Pisco estimates that between 2013 and 2015, about a third of the actual workforce of 6000 primary care physicians will retire[6].

In Portugal, specialist training in GP/FM was increased from three to four years in 2009, and includes, apart from four different rotations in primary health care, compulsory hospital rotations in gynaecology and obstetrics, paediatrics, mental health/psychiatry, and emergency medicine. It also caters for a number of elective rotations and short rotations aimed at the acquisition of specific skills[7]. Furthermore, there is a growing number of vocational trainees also enrolled in post-graduate studies, masters and doctoral degrees.

The author of this article informally surveyed a number of family doctors and vocational trainees through national mailing lists in Portugal and Spain (with around 600 and 450 members respectively), as well as most trainees and tutors from his health care centre, in order to gauge what doctors considered to be the most positive and negative aspects of the specialty. Two members of the Portuguese mailing list and three members of the Spanish mailing list replied. The most positive aspects included being the least hierarchical specialty, the large number of opportunities available, the holistic approach, the possibility of solving the great majority of health problems, the absence of limits in terms of knowledge, and the greater compatibility with family life.

The most negative aspects pointed out were the system of fixed salaries and lack of incentives in place, the "existential crisis" the specialty is going through in Europe, the greater tendency towards professional stagnation and isolation, the lack of time during appointments, and the limitations to carrying out technical procedures.

At a European level, there is a lack of harmonization, as GP/FM is considered a specialty in most but not all European countries[8,9]. Still, there is a European academic and scientific society of GP/FM (WONCA Europe), and even others for primary care research (EGPRN - European General Practice Research Network), teaching (EURACT - European Academy of Teachers in General Practice) and quality (EquiP - European Association for Quality in General Practice/Family Medicine). There is also a political organization, called UEMO, which stands for European Union of General Practitioners, whose rotational presidency is currently held by Portugal, as well as an organization for trainees and young general practitioners called WONCA Europe Working Group for Young and Future General Practitioners (also known as Vasco da Gama Movement).

Denmark, The Netherlands, UK, and Spain have been considered for a long time the leading nations in GP/FM[10]. On the other hand, countries like Portugal and Spain have been championing innovative and pioneering initiatives in the virtual world like the Virtual Congress of General Practice/Family Medicine and the Second Life Family and Community Medicine International Congress, respectively.

Contrarily to what many people think, GP/FM is alive and well, but there's still a long way to go. There are new challenges, new barriers, new threats, but also new opportunities.

In order to succeed, the 21^st ^century GP/family physician must be self-confident and believe in oneself, must know how to cooperate with other health care professionals and levels of care, must know how to think in terms of both the individual patient and in terms of the community the patient belongs to, must practice with human and technical quality, must fight against the abuse of technology and the fragmentation of healthcare, and is expected to be prestigious, competent and polyvalent[5].

General Practice/Family Medicine has great potential for scientific development in the 21^st ^century, and in the case of the Portuguese reality, has been growing frankly in terms of quality and quantity, fuelled in large part thanks to the massive organizational reform of primary health care in Portugal and the fact that General Practice/Family Medicine is already a first choice for many junior doctors in Portugal[11].

It is already widely acknowledged that the countries with the best health systems are usually those with a strong primary care infrastructure: they tend to have healthier populations, fewer health-related disparities and lower overall costs for health care[12]. If that is the case, then what is the matter?

The answer is not straightforward. Making General Practice/Family Medicine more attractive is surely part of the equation, but it is also undoubtedly important not to neglect the importance of improving payment schemes, as family physicians tend to earn less than hospital specialists, as well as of investing in primary care infra-structure and organization, like high quality health information technology[13].

It is also important not to forget that there are significant differences between different European countries in terms of the existence of structured curricula for vocational training, funding for structured vocational training, professional recognition of trainers, equity of salary between hospital and GP trainees, and these are issues that must also be addressed in order to increase the appeal of GP/FM[8].

The struggle for European recognition is ongoing, as general practice/family medicine aims to establish itself as the paradigm of 21^st ^century medicine.

## Competing interests

Tiago Villanueva is a member of the Organizing Committee of the Second Virtual Congress of General Practice/Family Medicine.
